# Association Between Rheumatic Autoantibodies and Immune-Related Adverse Events

**DOI:** 10.1093/oncolo/oyac252

**Published:** 2023-01-03

**Authors:** Kristen Mathias, Sherin Rouhani, Daniel Olson, Anne R Bass, Thomas F Gajewski, Pankti Reid

**Affiliations:** Department of Medicine, University of Chicago Medical Center, Chicago, IL, USA; Department of Medicine, Section of Hematology Oncology, Chicago, IL, USA; Department of Medicine, Section of Hematology Oncology, Chicago, IL, USA; Department of Medicine, Division of Rheumatology, Hospital for Special Surgery, Weill Cornell Medicine, New York, NY, USA; Department of Medicine, Section of Hematology Oncology, Chicago, IL, USA; Committee on Clinical Pharmacology and Pharmacogenomics, Chicago, IL, USA; Committee on Clinical Pharmacology and Pharmacogenomics, Chicago, IL, USA; Department of Medicine, Section of Rheumatology, University of Chicago Medical Center, Chicago, IL, USA

**Keywords:** autoantibodies, immune-related adverse events, immune checkpoint inhibitors, arthritis, cyclic citrullinated peptide, rheumatoid factor

## Abstract

**Background:**

Side effects of immune checkpoint inhibitors (ICIs), called immune-related adverse events (irAEs), closely resemble primary autoimmune or rheumatic diseases. We aimed to understand the clinical utility of rheumatic autoantibodies (rhAbs) for diagnosing irAEs.

**Patients and Methods:**

Patients without pre-existing autoimmune disease (pAID) who had cancer treated with ICI(s) treatment from 1/1/2011 to 12/21/2020 *and* a rhAb checked were retrospectively identified. Logistic regression assessed associations between autoantibodies and irAEs, cancer outcome, and survival. Specificity, sensitivity, and positive/negative predictive values (PPV, NPV) were estimated for key rhAbs and ICI-arthritis. Kaplan-Meier analyzed objective response rate (ORR) and overall survival (OS).

**Results:**

A total of 2662 patients were treated with≥1 ICIs. One hundred and thirty-five without pAID had ≥ 1 rhAb tested. Of which 70/135(52%) were female; median age at cancer diagnosis was 62 years with most common cancers: melanoma (23%) or non–small cell lung cancer (21%), 96/135 (75%) were anti-PD1/PDL1 treated. Eighty had a rhAb ordered before ICI, 96 after ICI, and 12 before and after. Eighty-two (61%) experienced an irAE, 33 (24%) with rheumatic-irAE. Pre-ICI RF showed significant association with rheumatic-irAEs (OR = 25, 95% CI, 1.52-410.86, *P* = .024). Pre– and post–ICI RF yielded high specificity for ICI-arthritis (93% and 78%), as did pre– and post–ICI CCP (100% and 91%). Pre–ICI RF carried 93% NPV and pre–ICI CCP had 89% PPV for ICI-arthritis. No variables were significantly correlated with ORR. Any-type irAE, rheumatic-irAE and ICI-arthritis were all associated with better OS (*P* = .000, *P* = .028, *P* = .019).

**Conclusions:**

Pre–ICI RF was associated with higher odds of rheumatic-irAEs. IrAEs had better OS; therefore, clinical contextualization for rhAbs is critical to prevent unnecessary withholding of lifesaving ICI for fear of irAEs.

## Teaser

Side effects of immune checkpoint inhibitors, immune-related adverse events (irAEs), closely resemble primary autoimmune or rheumatic diseases. This article reports on the clinical utility of rheumatic autoantibodies for diagnosing irAEs.

**Implications for Practice**

For patients without preexisting autoimmune diseases, rheumatoid factor (RF) positivity prior to immune checkpoint inhibitor initiation (pre-ICI) is associated with a 25-fold higher odds of developing a rheumatic immune-related adverse event (OR = 25, 95% CI, 1.52-410.86, *P* = .024). Pre–ICI RF and pre–ICI cyclic citrullinated peptide (CCP) positivity have high specificity for ICI-arthritis. Pre–ICI CCP positivity indicates an 89% likelihood of arthritis development after ICI initiation. The authors recommend dedicated investigation of joint symptoms prior to ICI initiation and checking RF and CCP antibodies prior to ICI in patients with risk factors or symptoms of inflammatory arthritis; however, pre–ICI serologic positivity by itself should not preclude patients from receiving treatment with ICIs.

## Background

Immune checkpoint inhibitors (ICIs) have transformed survival outcomes for many patients with advanced cancers. ICIs target key proteins in immune checkpoint pathways that normally suppress the host immune response against tumor cells. As the immune response becomes unleashed against cancer cells, it also can mount inflammatory response against normal tissues. These toxicities can affect any organ system and are broadly referred to as immune-related adverse events (irAEs) [1, 2].

The pathophysiology of irAEs involves T–cell activation and, in some cases, B–cell-mediated production of autoantibodies [3]; however, the exact role of humoral autoimmunity in the pathophysiology of many irAEs is incompletely understood. IrAEs may induce changes in B cells that predispose patients to autoimmunity [4]. Correspondingly, multiple clinical studies have demonstrated organ-specific autoantibodies in patients with irAEs after ICI treatment [5-8].

Studies analyzing rheumatic-irAEs have found that the frequency of autoantibody positivity is generally lower than in patients with primary autoimmune diseases [3, 9, 10]. In patients with established ICI-arthritis, RF and/or CCP autoantibodies may be detectable in up to 11% of patients [11]. Specifically in regard to pre-ICI treatment autoantibodies, a 2021 review of over 500 publications presented studies that suggested that positive pre-ICI serologies may be biomarkers for irAE incidence[11]. This idea was supported in original work by Gowen et al, Toi et al, and Tahir et al [12-14]. Together, these findings bolster the hypothesis that baseline or pre-ICI imbalance of humoral immunity may play a prominent role in the later development of ICI toxicities. However, evidence for the predictive value of autoantibodies is mixed. In a prospective study, Ghosh et al showed that patients who developed organ-specific irAEs have fewer autoantibodies at baseline and a greater change in antibody concentration compared to patients who did not have an organ-specific irAE [15]. De Moel et al also found an association between antibody seroconversion and irAEs but no significant correlation with pre-treatment antibodies and irAEs [8].

Given this uncertainty surrounding autoantibodies and irAEs, there is no reliable guidance on the utility of autoantibodies in clinical practice. In 2021, the European League Against Rheumatism (EULAR) commissioned a task force to comment on the diagnosis and management of irAEs [16]. Regarding the ordering of autoantibodies, they concluded that it is unnecessary to test every patient receiving ICIs for autoantibodies but appropriate if the patient has rheumatologic or “systemic symptoms of unclear etiology.” This recommendation was grade D and based on level 5 evidence [16]. Our study aimed to investigate the clinical utility of pre– and post-ICI rhAbs in context of all-–type irAEs, rheumatic-irAEs, and ICI-arthritis.

## Methods

### Study Design

This was a retrospective observational study of patients who received at least one cycle of Program Death-1/Program Death-Ligand 1 (PD-1/PD-L1) immunotherapy (nivolumab, pembrolizumab, atezolizumab, or durvalumab) and/or Cytotoxic T-Lymphocyte-Associated Protein 4 (CTLA-4) inhibitor (ipilimumab) immunotherapy at University of Chicago Medical Center between January 1, 2011 and December 21, 2020, and who had at least one rhAb checked during any time in the course of their clinical care. Patients with diagnosis of one or more pre-existing autoimmune diseases (pAIDs) were analyzed separately (Supplementary Table S1). Collection of data for this study was approved by the University of Chicago Medical Center Institutional Review Board.

### Data Extraction

Charts were filtered by autoantibody results and ICI use then reviewed independently by 3 physicians (K.M., M.V.L., and P.R.). Clinical data were retrospectively extracted from institutional electronic medical records. Information on demographics (age, gender, and race), duration of ICI treatment, best response to ICI, type of irAE while on ICI, and the presence or absence of a pAID was collected. Characteristics of the autoantibody orders were additionally recorded, including information about the provider who ordered the lab and their clinical reasoning for checking it.

Details regarding irAE characteristics were extracted from clinical annotations and patients were deemed to have an irAE as reported and described by clinical provider(s) in the electronic medical record. IrAE severity was defined by the Common Terminology Criteria for Adverse Events version 5 (CTCAEv5) criteria. Tumor response to ICI was assessed by the Response Evaluation Criteria in Solid Tumors version 1.1 (RECIST v 1.1) criteria based on the best overall response since ICI therapy initiation.

### Autoantibody Testing

RhAbs included in our study were as follows: antinuclear antibody (ANA), rheumatoid factor (RF), cyclic citrullinated protein (CCP), Sjögren’s-syndrome-related antigen A (SSA) and B (SSB) autoantibodies, double-stranded DNA (dsDNA), antineutrophil cytoplastic antibodies (ANCA), and angiotensin converting enzyme (ACE) as well as antibodies to smith antigen, ribonucleoprotein, topoisomerase I (anti-Scl-70), and Jo-1 or myomarker panel (includes following antibodies: Mi-2, Ku, PM-Scl100, PL-7, PL-12, EJ, OJ, SRP, TIF-1 gamma, NXP2). ANA testing was conducted by a clinical laboratory improved amendments-certified lab via indirect immunofluorescence method. ANA ≥ 1:80 was considered positive. RF ≥ 14 [iU]/mL was considered positive and CCP ≥ 3.0 U/mL was considered positive. Antibodies measured prior to ICI initiation were deemed “pre-ICI” and after ICI initiation were referred to as “post-ICI.”

### Statistical Analysis

Categorial data were reported as frequencies and percentages. Continuous variables were reported as median along with the range. Logistic regression model was used to evaluate the relationship between irAE development and autoantibody results as well as tumor outcome while adjusting for significant covariates. Missing data were accounted for by way of listwise deletion. We radiographically defined complete response (CR), partial response (PR), stable disease (SD), progression disease (PD) with reference to the Response Evaluation Criteria in Solid Tumors (RECIST), version 1.1 [17]. The objective response rate (ORR) was defined as CR plus PR. Overall survival (OS) was defined as the time interval from the start of ICIs therapy until death or last follow-up and was calculated using the Kaplan-Meier method. The log-rank test was applied to test for statistical significance. Specificity, sensitivity, and predictive values were estimated for key rhAbs and checkpoint inhibitor-associated arthritis (ICI-arthritis). All data were analyzed using STATA 15.1. Tests were performed at a significance level of *α* = 0.05 and values were considered statistically significant if *P*-value was less than 0.05.

## Results

### Patient Population

In total, 2662 patients received one or more ICIs during their clinical care between January 2011 and December 2020 at the University of Chicago Medical Center. From this group, 152 patients had at least one rhAb ordered at any time during the course of their clinical care. Seventeen of the 152 patients carried a diagnosis of an autoimmune disease prior to ICI initiation and were analyzed separately (Supplementary Table S1). Ultimately, 135 patients without pAIDs were included in the main analysis (Table I).

**Table 1.** Study population.

Total patients treated with ICI and had ≥1 rhAb checked and without pre-existing autoimmune disease	135
Gender	
Female	70 (52%)
Male	65 (48%)
Age in years at cancer diagnosis, median (range)	62 (16-94)
Race	
White	83 (62%)
Black/African American	37 (27%)
Asian/Mideast Indian	5 (4%)
Hispanic	2 (1%)
American Indian/Alaskan Native	1 (1%)
More than one race	6 (4%)
Patient declined	1 (1%)
Primary malignancy	
Melanoma	31 (23%)
Non-small cell lung cancer	28 (21%)
Genitourinary cancers	16 (12%)
Small cell lung cancer	13 (10%)
Head and neck squamous cell carcinoma	8 (6%)
Hepatocellular carcinoma	7 (5%)
Other	32 (24%)
Immunotherapy	
CTLA-4i	14 (10%)
PD-1/PD-L1i	96 (71%)
Combination	25 (19%)
Immune-related adverse events	
Any-type irAE	82 (61%)
Rheumatic irAE	33 (24%)
ICI-arthritis	28 (21%)
Severity based on CTCAEv5 for irAEs (82)	
Grade 1	11 (13%)
Grade 2	24 (29%)
Grade 3	33 (40%)
Grade 4	14 (16%)
Grade 5	2 (2%)
Anytime autoantibody positivity	95 (70%)
Pre-ICI autoantibody positivity (positive/checked (%))	
Any type autoantibody	57/80 (71%)
ANA	51/68 (75%)
RF	8/40 (20%)
CCP	2/15 (13%)
SSA, SSB	2/17 (12%)
dsDNA	1/51 (2%)
ANCA	0/14 (0%)
Post-ICI autoantibody positivity (positive/checked (%))	
Any type autoantibody	60/96 (63%)
ANA	54/81 (67%)
RF	17/70 (24%)
CCP	5/47 (11%)
SSA, SSB	3/51 (6%)
dsDNA	1/68 (1%)
ANCA	2/31 (6%)

^*^Rheumatic irAE count does not include PAD disease flares.

Abbreviations: CTLA-4i, cytotoxic T-lymphocyte-associated protein 4 inhibitor; PD-1/PD-L1i, program death-1/program death-ligand 1 inhibitor; CTCAEv5, Common Terminology Criteria for Adverse Events version 5; ANA, antinuclear antibody; RF, rheumatoid factor; CCP, Cyclic citrullinated protein; SSA, Sjögren’s-syndrome-related antigen A autoantibody, SSB, Sjögren’s-syndrome-related antigen B autoantibody dsDNA, double-stranded DNA; ANCA, antineutrophil cytoplastic antibodies.

### Patients with Pre-existing Autoimmune Disease

Seventeen patients (11%) of 152 patients in our cohort had a diagnosis of autoimmune disease prior to ICI initiation or pre-existing autoimmune disease (pAID) which are discussed here separately (Supplementary Table S1). These 17 patients with pAID were excluded from our final analysis of the 135 patients who did not have pAIDs. The most common pAID diagnoses were rheumatoid arthritis (RA) (*n* = 5) or psoriasis/psoriatic arthritis (PsO/PsA) (*n* = 5). None of the patients with pAIDs were reported to have clinically active disease prior to starting immunotherapy. None of these patients continued their steroid-sparing immunomodulating agents after start of cancer immunotherapy. Six patients (35%) experienced a flare of their pAID (4 with RA flare, 1 with PsO/PsA flare, and 1 with celiac flare). Four patients (24%) suffered from a de novo irAE (1 with hepatitis and colitis, 1 with hepatitis only, 1 with colitis only, and 1 with dermatitis). To test the sensitivity and specificity of rhAbs for predicting de novo autoimmunity and irAEs, we excluded this cohort of patients with pAID from subsequent analyses.

### Patient Characteristics

Of the 135 patients without pAID, 31 had melanoma (23%), 28 had non–small cell lung cancer (21%), 16 had genitourinary cancer (12%), 13 had small cell lung cancer (10%), and 39 had other cancers (Table I). Most patients received PD1/PDL1 antibody monotherapy (71%), with the remaining patients receiving either CTLA-4 antibody monotherapy (10%) or combination therapy (19%).

### Characteristics of Rheumatic Autoantibodies

In our cohort of 135 patients without pAID, the most commonly positive autoantibodies were ANA followed by RF (Table I). Eighty patients had a rhAb checked prior to ICI initiation (pre-ICI) with 57 (71%) positive results. Ninety-six patients had an autoantibody checked after start of ICI therapy (post-ICI) with 60 (63%) positive. The 2 most common reasons for providers to check autoantibodies were arthralgias and liver function test abnormalities (Supplementary Table S2). Providers who ordered the pre– and post-ICI antibodies are reflected in Supplementary Fig. S2.

Of the 135 patients without pAIDs, 12 patients had at least one of the same rhAbs checked both prior to and after ICI initiation (pre-ICI and post-ICI, respectively) (Supplementary Table S3). Of these 12 patients, 6 patients had an increase in their antibody titers, 3 had a decrease, and 3 did not have a significant change. Regardless of change in serologies, all of the patients who had one or more of the same rhAbs checked before and after ICI experienced an irAE.

### Immune-Related Adverse Events Features

**Figure 1.**
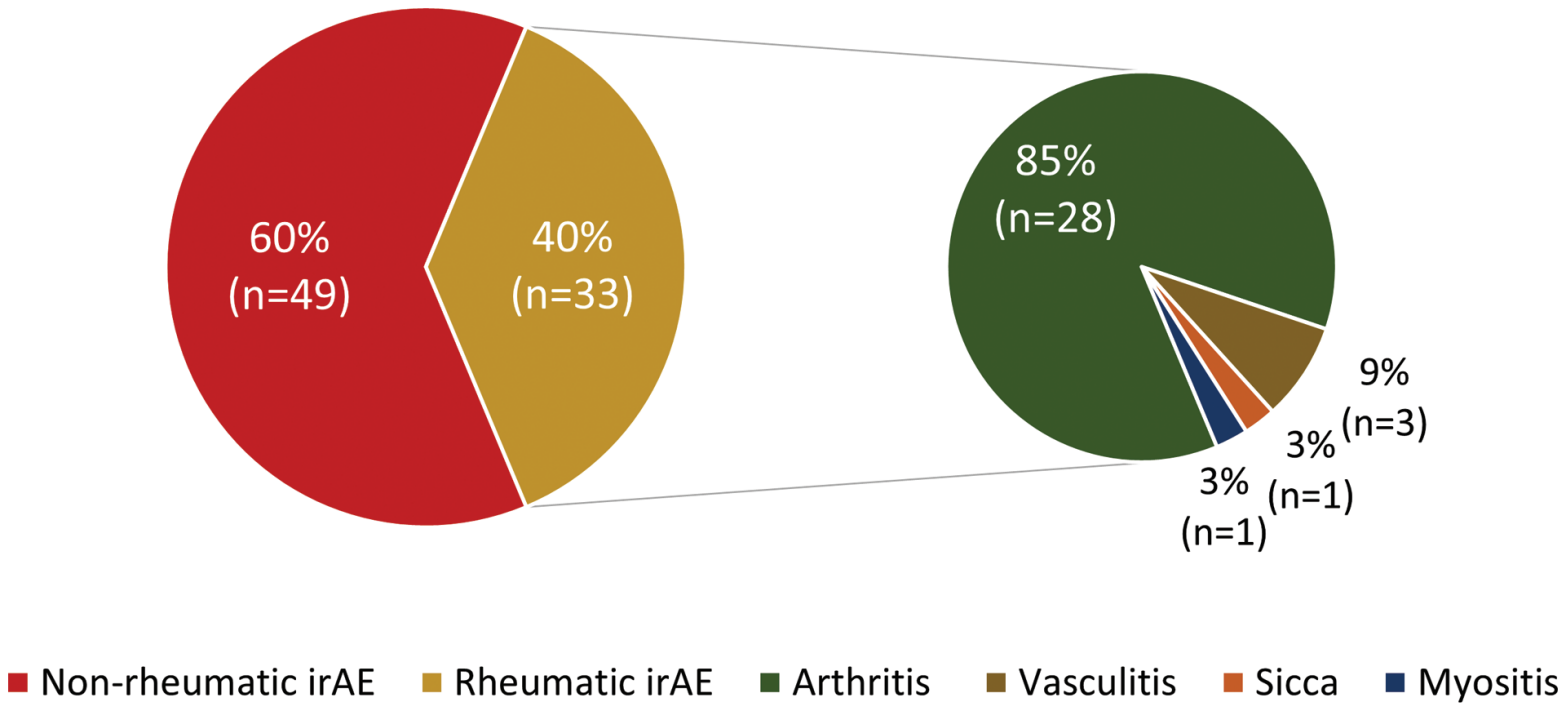
Immune-related adverse events subtypes: Our cohort of patients included patients who were treated with immune checkpoint inhibitor, had a rheumatic autoantibody ordered at some point in their clinical care, and did not carry a diagnosis of autoimmune disease prior to ICI initiation. A total 82/135 (61%) patients in our cohort of patients experienced an immune-related adverse event (irAE). Of these, 33/82 (38%) were rheumatic-irAEs as reflected in this pie chart.

Eighty-two of the 135 patients (61%) without pAID developed irAEs (Table 1). Over half of the irAEs (49 of 82) were classified as CTCAE grade 3 or higher (Table 1). A total of 33 patients (24%) developed rheumatic irAEs: 28 had ICI-arthritis, 3 with ICI-vasculitis, 1 ICI-myositis, and 1 ICI-sicca (Fig. 1).

### Correlates of Any-Type irAEs, Rheumatic irAEs

Significant correlates of irAEs are presented in Table 2. After adjusting for significant covariates, there was a significant positive correlation with that of any-time RF positivity and rheumatic-irAEs (OR 3.79, 95% CI, 1.20-11.96, *P* = .023) but a trend toward a negative correlation between any-time ANA positivity and rheumatic-irAEs (OR 0.42, 95% CI, 0.17-1.02, *P* = .055) (Table 2). Having pre-ICI RF positivity in particular was significantly associated with rheumatic-irAE development compared to patients with pre-ICI RF negativity (OR 25.00, 95% CI, 1.52-410.86, *P* = .024). No significant association was found between any post-ICI autoantibody positivity with that of any-type irAEs or rheumatic-irAEs.

**Table 2.** Correlates of any-type irAEs, rheumatic irAEs, and objective response rate.

Covariates	Any-type irAEs		Rheumatic irAEs	
	OR (95% CI)^a^	*P*-value^a^	OR (95% CI)^a^	*P*-value^a^
Age at cancer diagnosis	0.99 (0.97-1.02)	.521	1.00 (0.98-1.03)	.829
Sex, male	1.38 (0.69-2.78)	.941	0.88 (0.41-1.90)	.738
Race		.763		.144
Tumor type		.324		.161
Type of ICI		.959		.877
Any-time serologies				
Any-time ANA positivity	0.06 (0.27-1.41)	.247	0.42 (0.17-1.02)	.055
Any-time RF positivity	1.80 (0.46-7.01)	.394	3.79 (1.20-11.96)	.023
Pre-ICI serologies				
Pre-ICI Any rhAb positivity	0.96 (0.34-2.67)	.938	0.97(0.22-4.33)	.973
Pre-ICI ANA positivity	1.13 (0.34-3.74)	.847	1.89(0.20-17.65)	.576
Pre-ICI RF positivity	2.91 (0.23-36.16)	.406	25.00(1.52-410.86)	.024
Post-ICI serologies				
Any post-ICI rhAb	0.44 (0.11-1.74)	.244	0.53(0.21-1.31)	.167
Post-ICI ANA positivity	0.31 (0.06-1.55)	.155	0.44(0.16-1.20)	.110
Post-ICI RF positivity	0.75 (0.13-4.24)	.745	2.04(0.58-7.10)	.264
Tumor outcome				
Objective response rate^b^	4.34 (1.54-12.26)	.006	1.98 (0.80-4.88)	.140

^a^Table reflects results from multivariate analysis, controlled for significant covariates on univariate analysis (at *P* < .05 level) as applicable.

^b^According to the Response Evaluation Criteria in Solid Tumors version 1.1 (RECIST v 1.1) criteria based on the best overall response since ICI therapy initiation.

All 9 patients tested had negative CCP—immeasurable due to collinearity.

Only 3 patients of the 42 tested were positive for CCP: 1 had myositis, 1 with arthritis, and 1 with pneumonitis.

Abbreviations: ANA, anti-nuclear autoantibody; CI, confidence interval; ICI, immune checkpoint inhibitor; irAEs, immune-related adverse events; OR: odds ratio; rhAb: rheumatic autoantibody; RF: rheumatoid factor.

### Correlates with Tumor Outcome and Survival

**Figure 2.**
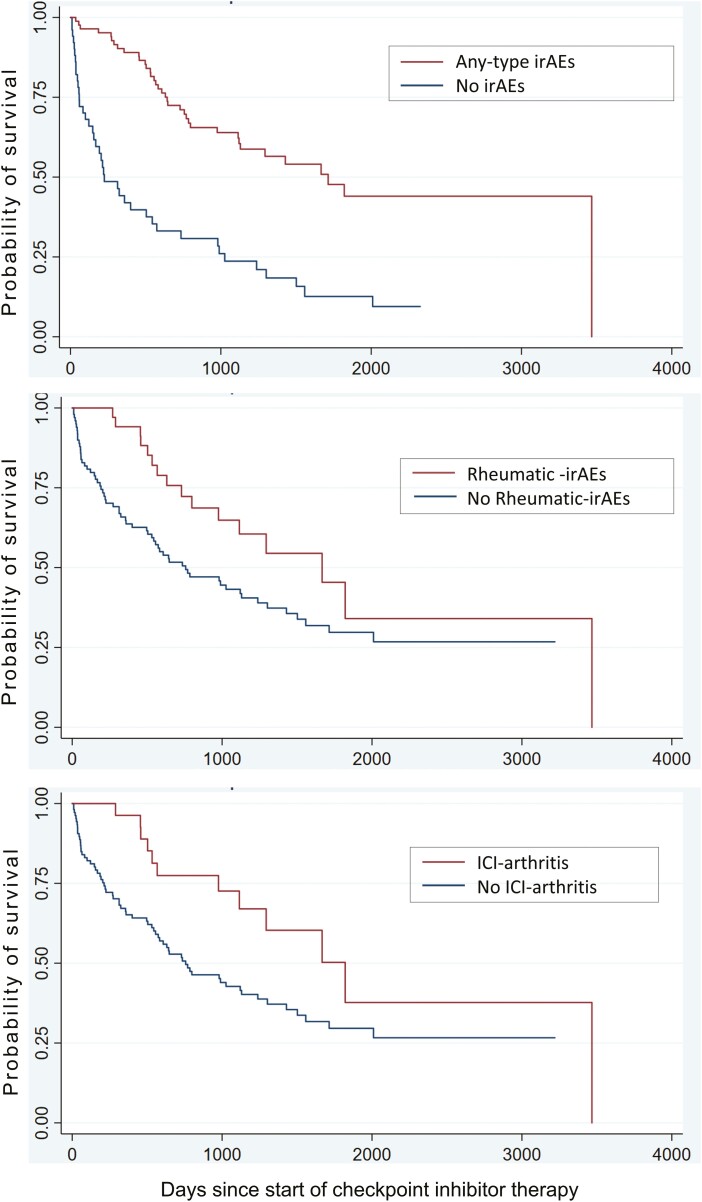
Correlates of overall survival: any-type irAEs showed a significant correlation with OS (*P* = .000) as did rheumatic-irAEs (*P* = .028) and ICI-arthritis (*P* = .019). No serologic analyses were significantly correlated with OS.

Significant correlates of ORR are summarized in Supplementary Table S4. Any-type irAE presence was associated with ORR (OR 4.34, 95% CI, 1.54-12.26, *P* = .006), but no other variables were significantly correlated with ORR. Kaplan-Meier curves of OS for any-type irAEs, rheumatic-irAEs and ICI-arthritis are presented in Fig. 2. Other correlates of OS included age at cancer diagnosis, tumor type, any-type irAE, and ICI-arthritis as tabulated in Supplementary Table S5. Any-type irAEs also showed a significant correlation with OS (*P* = .000) as did rheumatic-irAEs (*P* = .028) and ICI-arthritis (*P* = .019). No serologic analyses were significantly correlated with OS.

### RhAb Specificity, Sensitivity, and Predictive Values for ICI-Arthritis

Table 3 reflects values for specificity, sensitivity, positive, and negative predictive values (PPV and NPV) as well as prevalence of ICI-arthritis within the group of patients without pAID with respective rhAb testing. When looking at cases of ICI-arthritis, pre-ICI RF and pre-ICI CCP yielded high specificities (93% and 100%, respectively) but low sensitivities (33% and 0%, respectively) for ICI-arthritis (Table 3). Pre-ICI RF showed NPV of 93% with prevalence of ICI-arthritis of 10% in the patient group with pre-ICI RF tested. Pre-ICI CCP showed a PPV value of 89% with prevalence of ICI-arthritis of 10% in the group of patients with pre-ICI CCP tested. The sensitivity, specificity, and predictive values of post-ICI autoantibodies (any rhAb, RF, and CCP) are shown in Table 3. All PPVs and NPVs for these post-ICI serologic tests were less than 60%, with prevalence of ICI-arthritis that ranged from 33% in post-ICI any rhAb-tested group to 48% in post-ICI CCP-tested group (Table 3).

**Table 3.** Sensitivity and specificity of Rheumatic autoantibodies for ICI-arthritis.

	Sensitivity (%)	Specificity (%)	PPV (%)	NPV (%)	Prevalence^a^
Any pre-ICI RhAb	50.00	31.15	6.67	86.36	9%
Pre-ICI RF	33.33	92.59	33.33	92.59	10%
Pre-ICI CCP	0.00	100.00	88.89	Incalculable	10%
Any post-ICI RhAb	53.57	29.82	27.27	56.67	33%
Post-ICI RF	19.23	78.38	38.46	58.00	41%
Post-ICI CCP	5.00	90.91	33.33	51.28	48%

^a^Prevalence of ICI-arthritis.

Abbreviations: RhAb, rheumatic autoantibody; ICI-arthritis, immune-checkpoint inhibitor-induced arthritis; RF, rheumatoid factor; CCP, cyclic citrullinated peptide; PPV, positive predictive value; NPV, negative predictive value.

## Discussion

In this study of 135 ICI-treated patients with one or more rheumatic autoantibody tested and without pAID, we found that pre-ICI RF positivity was significantly associated with higher odds of rheumatic-irAE development. Rheumatic-irAEs and ICI-arthritis were also correlated with better OS. With a focus on ICI-arthritis, both pre– and post-ICI RF and CCP showed high specificity for ICI-arthritis. Pre-ICI RF additionally showed high NPV for ICI-arthritis and pre-ICI CCP showed a high PPV for ICI-arthritis.

Our analysis highlights the significance of pre-ICI RF for rheumatic-irAEs and ICI-arthritis and CCP for ICI-arthritis in a population without a diagnosis of pre-existing autoimmune disease. While any-type (unselective) pre-ICI rhAb positivity was not significantly associated with any-type irAEs or rheumatic-irAEs, pre-ICI RF was associated with a 25-fold higher odds of rheumatic-irAE development (OR 25.00, 95% CI, 1.52-410.86, *P* = .024). Pre-ICI RF also showed 93% specificity for ICI-arthritis. Pre-ICI CCP positivity had 100% specificity for ICI-arthritis. While there was a relatively small number of patients in our study who had these rhAbs checked and these findings need to be replicated in larger studies, these findings are also supported by the literature [13]. Other groups have shown that asymptomatic patients with a pre-ICI positive CCP antibody had an increased incidence of ICI-arthritis, suggesting that ICIs may accelerate arthritis in patients with asymptomatic CCP seropositivity [18, 19]. Sensitivities for pre– or post-ICI RF or CCP were all low. It should be noted that retrospective studies (including ours) with patients who had pre-ICI autoantibody checked as part of clinical care are subject to selection bias where the antibodies were likely ordered *in the setting* of ongoing signs or symptoms. For this reason, we caution against indiscriminate autoantibody testing prior to ICI initiation. Instead, we recommend explicitly inquiring about inflammatory joint pain as part of the review of symptoms prior to ICI initiation and having a low threshold to involve rheumatology if signs or symptoms raise concern for pre-existing rheumatic disease.

Beyond sensitivities and specificities of pre-ICI RF and CCP for ICI-arthritis, the high NPV of pre-ICI RF positivity and high PPV of pre-ICI CCP may provide better clinical relevance of these tests. Of note, as predictive values are impacted by prevalence, it is important to know that the 10% prevalence of ICI-arthritis in our patient population (10% in the pre-ICI RF-tested group and 10% also in the pre-ICI CCP-tested group) was comparable to the previously-reported prevalence of 1%-7% ICI-arthritis in a 2017 review[20]. That being said, the high NPV of pre-ICI RF from our findings offers reassurance that if a patient had a negative pre-ICI RF, there is a high (93%) likelihood that that patient will not develop post-ICI inflammatory arthritis. This is clinically noteworthy as this can potentially help avoid inappropriate withholding of ICI therapy and/or possibly deleterious administration of systemic corticosteroids with their associated side effects. In the context of ICI treatment and unclear impact of systemic immunosuppression on that of ICI efficacy and tumor outcome, preventing inappropriate systemic immunosuppressive agents can help avert theoretic risk of harm from their administration [21-25].

The high PPV of pre-ICI CCP, on the other hand, allows minimization of false positive diagnoses of ICI-arthritis, for example a patient that is diagnosed as having an inflammation-driven arthritis when the patient actually has non-inflammatory/mechanical joint disease or periarticular pain. The high PPV of pre-ICI CCP means that if a patient had pre-ICI CCP checked and it resulted positive, there is high likelihood (approximately 89% likelihood) that a patient with worsening symptoms has ICI-arthritis after ICI initiation. While alternative causes such as metastatic disease or septic arthritis must still be ruled out, the positive pre-ICI CCP can lead to earlier evaluation and increased confidence in the diagnosis of ICI-arthritis. In clinical practice, then, it may be worthwhile to order pre-ICI autoantibodies and obtain timely, dedicated rheumatology evaluation for patients with features of inflammatory arthropathy or a family history of rheumatic disease. Even if these symptoms would not fit the diagnosis for a pre-existing rheumatic disease, the high PPV of pre-ICI CCP would raise higher concerns for arthritis after ICI. While we advise *against* withholding of ICI therapy for this patient population (with symptoms or multiple risk factors for inflammatory arthritis and pre-ICI CCP positivity), we do recommend early follow-up with Rheumatology after ICI start if there are worsening arthritis symptoms after ICI initiation.

The utility of pre-ICI rhAbs is greatest in the context of adequate clinical evaluation and when compared to post-ICI rhAbs. Not only could pre-ICI serologies be of help in toxicity monitoring after the start of ICI therapy but could be of assistance to compare to post-ICI serologic testing if signs and symptoms of autoimmune disease were to worsen. Notably, we did not find a statistically significant association with post-ICI autoantibody positivity alone and that of any-type irAEs or rheumatic-irAEs; and so, the post-ICI antibody levels may best be interpreted in reference to their change compared pre-ICI antibody levels. This was demonstrated by a prospective cohort study where a greater autoantibody fold change at 6 weeks compared to baseline portended a higher incidence of certain irAEs [15]. These findings build on a retrospective study that demonstrated a nonsignificant association between development of any autoantibodies and any-type irAEs [8].

While pre-ICI RF or CCP positivity should warrant close follow-up, pre-ICI serologic positivity by itself should not preclude patients from receiving treatment with ICIs. First, rheumatic autoantibodies have been reported in patients with malignancy (RF frequency up to 25% and ANA frequency up to 42%) and tend to be higher in the elderly (more commonly affected by cancer), and so isolated autoantibody positivity should not exclude patients from cancer immunotherapy [13, 26-33]. Another point to keep in mind when patients with rheumatic autoantibody positivity are considered for ICI treatment is that the rheumatic-irAEs are often non-fatal and treatable [34-37]. Next, in our analysis, pre-ICI RF positivity was positively correlated with rheumatic-irAE development and rheumatic-irAEs were also associated with better OS. This association between rheumatic-irAEs and improved cancer outcomes has been further supported by multiple studies in the past [36, 38]. And while our findings did not yield any significant associations between any serologic variables and ORR, this may be due to our sample size and warrants further investigation. Previous studies have also reported discrepancies in this association between pre-ICI antibody positivity and tumor outcome: a prospective study by Ghosh et al did not find any statistically significant association between baseline ANA, RF, or CCP positivity and PFS or OS [15]. but a retrospective study by Toi et al conferred a positive correlation between pre-ICI autoantibody positivity and higher ORR or longer PFS [13, 15].

Finally, an interesting finding in our results was that of a nearly significant *negative* association between any-time ANA positivity and rheumatic-irAEs (OR 0.42, 95% CI, 0.17-1.02, *P* = .055) but a significant positive association with any-time RF positivity and rheumatic-irAEs (OR 3.79, 95% CI, 1.20-11.96, *P* = .023). Since ANA is most commonly implicated in systemic lupus erythematosus (SLE) pathogenesis, this discordant serologic association seen in our cohort could be reflected by the phenotypic heterogeneity of rheumatic-irAEs described in literature: there is a predominance of inflammatory arthritis and rarity of connective tissue diseases such as SLE[34, 39-42]. This is in line with previous findings where authors have differentiated the B cell subtype (CD21^lo^ B cells) that may be the main driver of post-ICI toxicity from that of the B cell subtypes seen in SLE pathogenesis[4, 43]. Our conflicting correlations between RF positivity versus ANA positivity with rheumatic-irAEs suggest that not all rheumatic serologies can be treated equally; certain antibodies may pose a risk for rheumatic-irAEs (RF) while others may confer a protective effect (ANA). It is important to note that upon separating pre- and post-ICI testing, neither pre– nor post-ICI ANA was significantly associated with higher odds of rheumatic-irAE development and that our results differ from previous findings by Toi et al who found a *positive* association between ANA positivity and any-type irAEs.[13] All of these inconsistencies warrant further high-powered, prospective work to better assess serologic positivity and rheumatic-irAE development.

Our analysis does carry some limitations. Although the study examined a demographically varied population from a tertiary referral center, its retrospective design with lack of randomization of patients allows for potential unrecognized confounders. There is a selection bias as patients included in the analysis had a clinical indication for a provider to check an autoantibody, and thus had a higher pre-test probability of autoantibody positivity than the general population receiving ICIs. Also, given that this is a retrospective study, the diagnosis of rh-irAEs may be impacted by practitioner bias as they are able to see the serologic results at time of clinical assessment, but the assessments can be very helpful in further reinforcing the diagnosis when there is high clinical suspicion of ICI-arthritis. Finally, for some analysis, the number of patients with a particular autoantibody checked was low, so our study may have been underpowered. Larger multi-center retrospective studies and/or prospective studies evaluating the relationship between pre– and post-ICI serologic testing and irAE development and tumor outcome are needed.

## Conclusion

Our study underscores the potential relevance of pre-ICI serologic testing and provides certain clinical guidance for diagnosis of ICI-arthritis. Our findings reinforce the importance of earlier subspecialty referral to rheumatology in context of joint symptoms prior to ICI initiation and emphasize the need for higher vigilance for development of rheumatic-irAEs with pre-ICI RF positivity. Clinical contextualization of rheumatic autoantibody positivity is critical and autoantibody positivity alone should not prevent a patient from receiving necessary cancer treatment with ICI due to concern for potential immune-mediated ICI toxicities.

## Supplementary Material

oyac252_suppl_Supplementary_Material

## Data Availability

The data underlying this article will be shared on reasonable request to the corresponding author.
