# Supplementary material for: Association Between Rheumatic Autoantibodies and Immune-Related Adverse Events
Source: Oncologist. 2023 Jan 3;28(5):440–8. doi: 10.1093/oncolo/oyac252 (PMC10166164; doi:10.1093/oncolo/oyac252)
Supplement: oyac252_suppl_Supplementary_Material [file oyac252_suppl_supplementary_material.docx]

**SUPPLEMENTARY MATERIALS**

**Supplementary Table 1:** Pre-existing autoimmune diseases in the total patient population who had Immune checkpoint inhibitor treatment and one or more rheumatic-autoantibody checked at any time during their care (152 patients)

| Pre-existing autoimmune diseases: 17 of 152 (11%) | |
| --- | --- |
| Rheumatoid arthritis  Psoriasis or Psoriatic arthritis  Hashimoto thyroiditis  Systemic lupus erythematosus  Sjogren’s syndrome  Systemic sclerosis  Vasculitis  Sarcoidosis  Celia’s disease  Primary biliary cirrhosis | 5  3  2  1  1  1  1  1  1  1 |
| Pre-existing Autoimmune Disease flare  No flare | 6 (35%)  11 (65%) |

**Supplementary Table 2:** Reasons for autoantibody orders in patients with immune checkpoint inhibitor treatment and without pre-existing autoimmune diseases (135 patients)

**2a: Pre-ICI autoantibody orders**

|  | **Pre-ICI autoantibody order reasons (n=80)** |
| --- | --- |
| Arthropathy | 11 |
| Liver function tests abnormal/Hepatopathy | 10 |
| Nephropathy | 4 |
| Neuropathy | 3 |
| Myopathy | 3 |
| vasculopathy | 2 |
| Rash | 2 |
| Part of clinical trial | 2 |
| Hypercoagulability | 2 |
| Altered mental status | 2 |
| Fatigue | 1 |
| Alopecia | 1 |
| Pericardial effusion | 1 |
| Hyperglobulinemia | 1 |
| Nonspecific dyspnea | 1 |
| Stroke | 1 |
| Concern for SLE | 1 |
| Unclear from clinical notes | 32 |

**2b: Pre-ICI autoantibody orders**

|  | **Post-ICI autoantibody order reasons (n=96)** |
| --- | --- |
| Arthropathy | 28 |
| Liver function tests abnormal/Hepatopathy | 10 |
| Part of clinical trial? | 7 |
| Myopathy | 6 |
| Rash | 5 |
| Pneumonitis | 5 |
| Neuropathy | 4 |
| Myocarditis | 3 |
| Vasculopathy | 3 |
| Nephropathy | 2 |
| Encephalitis | 2 |
| Angioedema | 1 |
| Bell's palsy | 1 |
| Fever | 1 |
| Hemolytic anemia | 1 |
| Pericardial effusion | 1 |
| Adrenal insufficiency | 1 |
| Unclear from clinical notes | 15 |

**Supplementary Figure 2:** Providers ordering Pre-ICI and Post-ICI antibodies

**
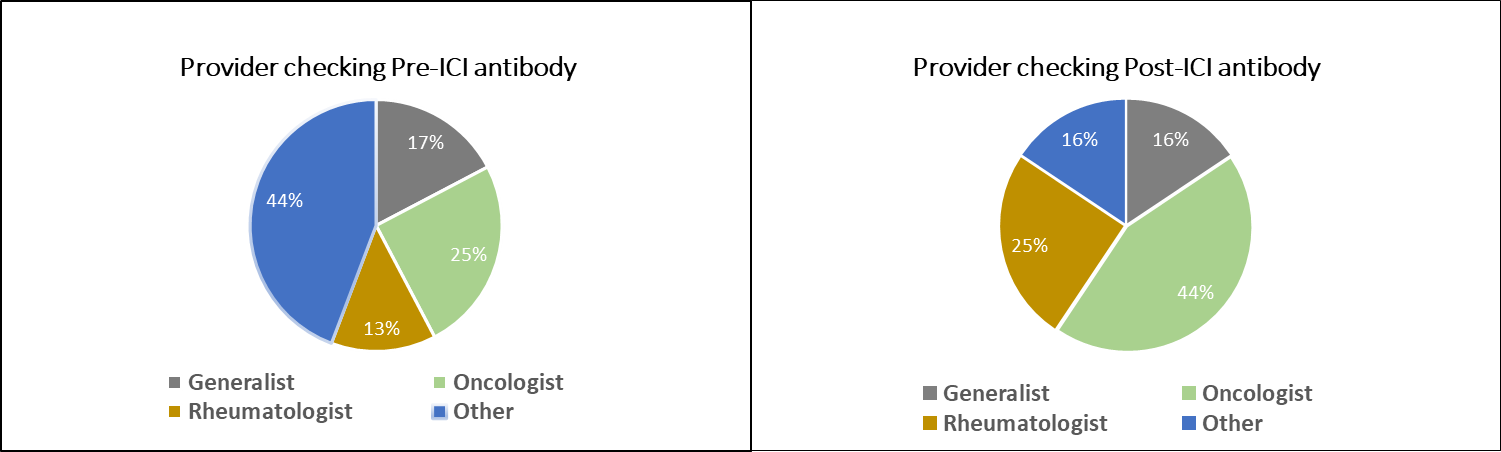
**

Pre-ICI : pre immune checkpoint inhibitor, Post-ICI: post immune checkpoint inhibitor, irAE: immune-related adverse event.

**Supplementary Table 3:** Characteristics of patients with the same rheumatic autoantibody checked prior to and after ICI start (and without pre-existing autoimmune disease)

| **Patient** | **Age at Cancer diagnosis** | **Legal Sex** | **Oncologic diagnosis** | **ICI Type** | **Tumor response** | **Pre-ICI RhAbs** | **Post-ICI RhAbs** | **Ab Change to** | **Type of IrAE** |
| --- | --- | --- | --- | --- | --- | --- | --- | --- | --- |
| 1 | 22.07 | M | Malignant melanoma | Combination | PD | Neg: ANA | ANA 1:80 | Increase  ANA seroconverted to positive | Hepatitis |
| 2 | 47.13 | F | Malignant melanoma | Combination | PD | Neg: ANA | ANA 1:320 | Increase  ANA seroconverted to positive | Hemolytic anemia |
| 3 | 60.33 | M | Malignant melanoma | PD1/PDL1i | PR | Neg: ANA | ANA 1:320 | Increase  ANA seroconverted to positive | Arthritis, myocarditis |
| 4 | 65.87 | F | Malignant melanoma | Combination | PR | Neg: ANA | ANA 1:2560 | Increase  ANA seroconverted to positive | Dermatitis, hepatotoxicity |
| 5 | 70.13 | M | Nonsmall cell lung carcinoma | PD1/PDL1i | PD | Neg: ANA, dsDNA | ANA 1:640  Neg: dsDNA | Increase  ANA seroconverted to positive | Hepatitis, colitis |
| 6 | 58.16 | F | Adrenocortical Carcinoma | Combination | PR | RF 9 | RF 32 | Increase  RF titer increase | Arthritis |
| 7 | 69.24 | F | Diffuse large B cell lymphoma | PD1/PDL1i | PD | ANA 1:320  Neg: ANCA | Neg: ANA, ANCA | Decrease  ANA seroconverted to negative | Hepatitis, cutaneous sarcoid |
| 8 | 50.66 | M | Hodgkins Lymphoma | PD1/PDL1i | SD | ANA 1:640  Neg: dsDNA | ANA 1:80  Neg: dsDNA | Decrease  ANA titer decrease | Anterior uveitis, hypothyroidism, bell's palsy |
| 9 | 68.65 | M | Urothelial cell carcinoma | PD1/PDL1i | SD | ANA 160  Neg: RF, SSA, SSB, ANCA, SCL70, RNP, Smith | ANA 1:80  Neg: RF, SSA, SSB, ANCA, SCL70, RNP, Smith | Decrease  ANA titer decrease | Arthritis, colitis, erythema multiforme, neuropathy, organizing pneumonia |
| 10 | 57.31 | M | Malignant melanoma | PD1/PDL1i | PD | ANA 1:320.  Neg: dsDNA, SSA, SSB | ANA 1:640  Neg: dsDNA, SSA, SSB | No significant change | Myositis |
| 11 | 61.63 | F | Squamous cell carcinoma | PD1/PDL1i | PD | Neg: SSA, SSB | Neg: SSA, SSB | No significant change | Cerebritis, possible gastritis |
| 12 | 15.59 | M | Hodgkins lymphoma | PD1/PDL1i | PD | ANA 1:320, 1:160.  Neg: dsDNA, SSA, SSB | ANA 1:80, 1:160  Neg: dsDNA, SSA, SSB | No significant change. ANA fluctuated 320, 160 --> fluctuated 160, 80 | Arthritis, sicca syndrome |

**Supplementary Table 4** Correlates of objective response rate

| **Covariates** | **Objective response rate** | |
| --- | --- | --- |
|  | **OR (95% CI) ^†^** | **P-value^†^** |
| Age at cancer diagnosis | 0.98 (0.95-1.00) | 0.083 |
| Sex, male | 0.75 (0.33-1.71) | 0.492 |
| Race |  | 0.918 |
| Tumor type |  | 0.553 |
| Type of ICI |  | 0.315 |
| **PRE-ICI SEROLOGIES** | | |
| Pre-ICI Any rhAb positivity | 0.27 (0.08-1.00) | 0.050 |
| Pre-ICI ANA positivity | 0.29 (0.06-1.35) | 0.115 |
| Pre-ICI RF positivity | 1.97 (0.00-27.35) | 1.000 |
| **POST-ICI SEROLOGIES** | | |
| Any Post-ICI rhAb | 2.16 (0.70-6.68) | 0.181 |
| Post-ICI ANA positivity | 0.80 (0.27-2.39) | 0.684 |
| Post-ICI RF positivity | 1.90 (0.54-6.69) | 0.319 |
| **IRAE DEVELOPMENT** | | |
| Any-type irAE | 4.34 (1.54-12.26) | 0.006 |
| Rheumatic-irAE | 1.98 (0.80-4.88) | 0.140 |
| *The cohort analyzed in this table excluded patients with pre-existing autoimmune disease.  ^†^Table reflects results from multivariate analysis, controlled for significant covariates on univariate analysis (at p<0.05 level) as applicable.  ANA: Anti-nuclear autoantibody, CI: Confidence interval, ICI: Immune checkpoint inhibitor, irAEs: Immune-related adverse events, OR: Odds ratio, rhAb: Rheumatic autoantibody, RF: Rheumatoid factor. | | |

**Supplementary Table 5:** Correlates of overall survival

| **Covariates** | **Overall survival** |
| --- | --- |
|  | **P-value^†^** |
| Age at cancer diagnosis | 0.0000 |
| Sex, male | 0.7116 |
| Race | 0.0538 |
| Tumor type | 0.0003 |
| Type of ICI | 0.0474 |
| **PRE-ICI SEROLOGY** |  |
| Pre-ICI Any rhAb positivity | 0.4835 |
| Pre-ICI ANA positivity | 0.3967 |
| Pre-ICI RF positivity | 0.2000 |
| **POST-ICI SEROLOGY** |  |
| Any Post-ICI rhAb | 0.7321 |
| Post-ICI ANA positivity | 0.8943 |
| Post-ICI RF positivity | 0.3957 |
| **IRAE DEVELOPMENT** | |
| Any type irAE | 0.0000 |
| Rheumatic irAE | 0.0278 |
| ICI-Arthritis | 0.0193 |
| ^†^Table reflects results from multivariate analysis, controlled for significant covariates on univariate analysis (at p<0.05 level) as applicable.  ANA: Anti-nuclear autoantibody, ICI: Immune checkpoint inhibitor, irAEs: Immune-related adverse events, rhAb: Rheumatic autoantibody, RF: Rheumatoid factor. | |
